# Hedgehog signalling does not stimulate cartilage catabolism and is inhibited by Interleukin-1β

**DOI:** 10.1186/s13075-015-0891-z

**Published:** 2015-12-24

**Authors:** Clare L. Thompson, Riana Patel, Terri-Ann N. Kelly, Angus K. T. Wann, Clark T. Hung, J. Paul Chapple, Martin M. Knight

**Affiliations:** Institute of Bioengineering and School of Engineering and Material Sciences, Queen Mary University of London, Mile End Road, London, E1 4NS UK; Center for Endocrinology, William Harvey Research Institute, School of Medicine and Dentistry, Queen Mary University of London, London, UK; Department of Biomedical Engineering, Columbia University, New York, NY USA; Kennedy Institute of Rheumatology, University of Oxford, Oxford, UK

**Keywords:** Osteoarthritis, Articular cartilage, Hedgehog signal transduction, Interleukin 1β, Cyclopamine, Indian hedgehog

## Abstract

**Background:**

In osteoarthritis, chondrocytes adopt an abnormal hypertrophic morphology and upregulate the expression of the extracellular matrix-degrading enzymes, MMP-13 and ADAMTS-5. The activation of the hedgehog signalling pathway has been established in osteoarthritis and is thought to influence both of these processes. However, the role of this pathway in the initiation and progression of osteoarthritis is unclear as previous studies have been unable to isolate the effects of hedgehog pathway activation from other pathological processes. In this study we test the hypothesis that hedgehog pathway activation causes cartilage degradation in healthy cartilage and in an *in vitro* model of inflammatory arthritis.

**Methods:**

Isolated articular chondrocytes from the bovine metacarpal-phalangeal joint were stimulated for up to 24 hours with the agonist, recombinant Indian hedgehog (r-Ihh). *ADAMTS-5* and *MMP-13* gene expression was quantified by real-time PCR. In addition, healthy bovine cartilage explants were treated with r-Ihh or the hedgehog antagonist, cyclopamine, and sGAG release into the media was measured over 72 hours. Studies were repeated using chondrocytes and cartilage explants from human knee joint. Finally, studies were conducted to determine the effect of hedgehog pathway activation on matrix catabolism in the presence of the pro-inflammatory cytokine, IL-1β.

**Results:**

Addition of r-Ihh activated hedgehog signalling, confirmed by upregulation of *Gli1* and *Ptch1* expression, but did not increase *ADAMTS-5* or *MMP-13* expression in bovine or human chondrocytes. Furthermore, r-Ihh did not induce sGAG release in healthy bovine or human cartilage explants. IL-1β treatment induced sGAG release, but this response was not altered by the stimulation or inhibition of hedgehog signalling. Hedgehog pathway activation was downregulated by IL-1β. Conversely, r-Ihh weakly suppressed IL-1β-induced *ADAMTS-*5 expression.

**Conclusion:**

Our results show for the first time that Indian hedgehog does not cause extracellular matrix degradation in healthy *ex vivo* cartilage or in the presence of IL-1β and that IL-1β downregulates Indian hedgehog induced signalling. Thus, we suggest reported hedgehog induced matrix catabolism in osteoarthritis must be due to its interaction with pathological factors other than IL-1β. Hence, hedgehog signalling and its downstream effects are highly context-dependent.

**Electronic supplementary material:**

The online version of this article (doi:10.1186/s13075-015-0891-z) contains supplementary material, which is available to authorized users.

## Background

Osteoarthritis (OA), characterised by the progressive degeneration of load-bearing diarthrodial joints, such as the knees and hips, is one of the leading causes of disability worldwide. In OA, the metabolic balance between extracellular matrix production and degradation is disrupted, leading to increased catabolic breakdown of the articular cartilage [1]. The mechanisms underlying OA are not fully understood, however, previous research indicates that disease progression is influenced by a combination of factors including injury, metabolism, age, and genetics [2]. Furthermore, strong evidence points to a significant inflammatory component to OA, involving cytokine cross-talk between chondrocytes, the synovium lining the joint capsule and the underlying bone [3].

The hedgehog signalling pathway has also been implicated in the pathogenesis of OA [4–6]. In articular cartilage, hedgehog pathway activation requires the binding of Indian hedgehog (Ihh) to its transmembrane receptor, Patched-1 (Ptch1). Ptch1 causes disinhibition of the transmembrane protein Smoothened (Smo), enabling its enrichment and activation in the primary cilium, a microtubule-based organelle found in most cells, including articular chondrocytes [7–9]. Smo indirectly activates the downstream Gli transcription factors, which in turn translocate to the nucleus to induce the expression of hedgehog target genes, such as *Ptch1* and *Gli1* [7, 8, 10, 11]. Hedgehog signalling regulates chondrocyte differentiation and proliferation during embryonic skeletal development and in the terminal growth plate [12, 13]. In OA, chondrocytes exhibit a phenotypic shift to a more terminal growth-plate-like state, adopting a similar hypertrophic morphology and upregulating the expression of hypertrophic markers, including type X collagen, matrix metalloproteinase (MMP)-13 and a disintegrin and metalloproteinase with thrombospondin motifs (ADAMTS)-5 [14]. Hedgehog pathway activation is thought to contribute to this process. Indeed, Lin et al. report that hedgehog pathway activation is increased in both human and mouse OA cartilage, with higher levels of hedgehog signalling correlating with increased disease severity [5]. Moreover, hedgehog blockade reduces the expression of OA-related genes, including *ADAMTS5* and *MMP13*, in mice with surgically induced OA and in human OA cartilage samples [5, 6]. Further evidence for the role of hedgehog pathway activation in OA stems from the increased expression of Indian hedgehog (Ihh) in cartilage and synovial fluid in human OA, with a 5.2-fold and a 1.71-fold increase, respectively, compared to their normal controls [4, 15].

Hedgehog pathway activation stimulates specific Gli2-mediated expression of runt-related transcription factor 2 (Runx2) [16]. Runx2 upregulates several matrix-degrading enzymes, including MMPs-1, 3, and 13 and ADAMTS-4 and ADAMTS-5, increases production of nitric oxide (NO) and prostaglandin E_2_ (PGE_2_) and upregulates the expression of type X collagen. The Ihh-Runx2 pathway thus represents a plausible mechanism by which the hedgehog pathway influences OA development as Runx2 functions upstream of many proteins identified in cartilage lesions in early OA [5, 17, 18].

While hedgehog pathway activation has been demonstrated to increase the likelihood of developing OA, the nature of these studies prevent analysis of hedgehog pathway activation in isolation from other processes that occur within the complex environment of the joint. In the current study we test if hedgehog pathway activation alone can stimulate cartilage degradation in healthy human and bovine articular cartilage samples. We report that pathway activation in response to Indian hedgehog (Ihh) does not influence the expression of *ADAMTS-5* or *MMP-13,* nor induce cartilage degradation, as measured by sulphated glycosaminoglycan (sGAG) release. The response to hedgehog pathway activation was also examined in an in vitro bovine model of inflammatory arthritis. We report that neither stimulation nor inhibition of the hedgehog pathway influenced cartilage degradation in the presence of IL-1β. Thus, we conclude that the catabolic effects of hedgehog signalling in OA must arise due to interaction with other physiological or pathological signalling pathways within the joint.

## Methods

### Reagents and media preparation

Chondrogenic media consisted of DMEM supplemented with 86 U/mL penicillin-streptomycin, 1.72 mM L-glutamine, 17.25 mM HEPES, 0.12 mg/mL ascorbate, and 10 % FBS (Sigma-Aldrich, Poole, UK). Recombinant human IL-1β (Peprotech, London, UK) stock solution consisted of 10 μg/mL in PBS containing 0.01 % BSA and 10 % FBS [19]. Recombinant human/mouse Ihh (r-Ihh) stock solution consisted of 200 μg/mL in PBS containing 0.01 % BSA (R&D Systems, Minneapolis, MN, USA). Cyclopamine stock solution was 10 μM in ethanol (EMD Millipore, San Diego, CA, USA). The effective doses and modes of action of the pharmaceuticals used in this study are listed in Table 1. The differences in the cyclopamine doses used for bovine and human samples were based on previous findings [20–24].

**Table 1:** The doses and modes of actions of hedgehog pharmaceuticals

Drug name	Molecular weight	Mechanism of action	Dose
r-Ihh	20,000 Da	Binds to Ptch1, causing Ptch1 inhibition and Smo activation [5, 41]	1 μg/mL
Cyclopamine	411.6 Da	Smo antagonist [20–24]	Bovine: 10 μM
			Human: 20 μM
KAAD-cyclopamine	698 Da	Smo antagonist [24, 42, 43]	1 μM
SANT-1	373.5 Da	Smo antagonist [20, 23, 44]	1 μM
GANT61	429.6 Da	Gli antagonist [39, 45, 46]	10 μM

*Ptch 1* patched 1, *r-Ihh* recombinant Indian hedgehog, *Smo* smoothened

### Chondrocyte isolation and culture

Primary bovine articular chondrocytes were isolated from the metacarpophalangeal joints of 1–2-year-old steers by enzymatic digestion as previously described [25]. Human primary articular chondrocytes from the tibial plateau of knee joints from three donors (one female and two male) aged 26–60 years, were purchased from Articular Engineering (Northbrook, IL, USA) and cultured without passage. Primary chondrocytes were plated at 80,000 cells per cm^2^ onto glass coverslips and cultured in chondrogenic media for 5 days (confluence) before treatment with 10 ng/mL IL-1β or r-Ihh (Table 1). After treatment, cultures were processed for real-time PCR as indicated below.

### Cartilage explant model

Full-thickness human articular cartilage explants (3 × 3 mm blocks) were harvested from macroscopically healthy regions of the tibial plateau of a male patient undergoing total joint arthroplasty for OA. The human tissue was obtained with informed patient consent and full National Health Service (NHS) ethical approval (East London and The City Research Ethics Committee - Ethics number: 07/Q0605/29). Full-thickness articular cartilage explants (5 mm diameter) were harvested metacarpophalangeal joints from 1–2-year-old steers as previously described [15]. Individual cartilage explants were then placed in 24-well plates and cultured in 1 mL of their respective experimental media as outlined in Table 1. The culture media was collected and replaced every 24 hours over the 72-hour culture period and stored at −20 °C for biochemical assay. In the IL-1β-treated groups, the media were further supplemented with 2.5 ng/mL or 10 ng/mL IL-1β during the first 24 hours in culture.

### Explant viability analysis

Following the 72-hour culture period, a cell viability assay was performed on representative bovine explants from all groups via LIVE/DEAD Cell Viability Assays (Molecular Probes, Paisley, UK) and imaged on a confocal microscope (Leica Microsystems, Wetzlar, Germany). The numbers of viable and dead cells in each field of view were counted using ImageJ (NIH, Bethesda, MD, USA) and used to calculate the percentage cell viability for each group.

### Sulphated glycosaminoglycan assay

Culture media was analysed using the 1,9-dimethylmethylene blue (DMMB) assay to determine the amount of sGAG released due to breakdown of the cartilage extracellular matrix [26]. Each sample was run in triplicate against a chondroitin sulfate standard curve (0–50 μg/mL). As all samples were of similar weights and dimensions, the sGAG release for each explant was normalized to the mean sGAG release of the untreated explants at the same time point to account for donor variability.

### RNA extraction, cDNA synthesis, and real-time PCR

RNA was extracted from isolated chondrocyte culture and converted to cDNA using the RNeasy and QuantiTect Reverse Transcription Kits (Qiagen, Manchester, UK), evaluated by gel electrophoresis, and quantified using spectrophotometry. Quantitative real-time PCR was performed as previously described [9]. The sequences of the primers used in this experiment are shown in Table 2. Fold-change in gene expression was calculated relative to 18S rRNA controls.

**Table 2:** Primer sequences for the genes measured in this experiment

Gene	Species	Sequence (5′-3′)
*Patched 1*	Bovine	F-ATGTCTCGCACATCAACTGG
		R-TCGTGGTAAAGGAAAGCACC
*Gli1*	Bovine	F-ACCCCACCACCAGTCAGTAG
		R-TGTCCGACAGAGGTGAGATG
*ADAMTS-5*	Bovine	F- GCCCTGCCCAGCTAACGGTA
		R- CCCCCGGACACACACGGAA
*MMP-13*	Bovine	F- CCCTTGATGCCATAACCAGT
		R- GCCCAAAATTTTCTGCCTCT
*18S rRNA*	Bovine	F- GCAATTATTCCCCATGAACG
		R- GGCCTCACTAAACCATCCAA
*ADAMTS-5*	Human	F- CCTTGTGGAAAGGGGAGAAT
		R- ACAGTGACGATAGGCAAACT
*MMP-13*	Human	F- AGCCACTTTATGCTTCCTGA
		R- TCAAACTGTATGGGTCCGTT
*18S rRNA*	Human	F- CGGCTACCACATCCAAGGAA
		R- AGCTGGAATTACCGCGGC

*ADAMTS-5* a disintegrin and MMP with thrombospondin motifs-5, *MMP-13* matrix metalloproteinase-13, *18S rRNA* 18S ribosomal RNA

### Statistical analysis

Statistical differences in gene expression, sGAG and cell viability were measured using two-way analysis of variance (ANOVA) with Tukey’s post hoc comparison (GraphPad, La Jolla, CA, USA). The threshold for statistical significance was set at *P* <0.05. All values are displayed as mean ± standard deviation.

## Results

### R-Ihh activates the hedgehog pathway but does not stimulate ADAMTS-5 and MMP-13 expression in bovine articular chondrocytes

**Fig. 1:**
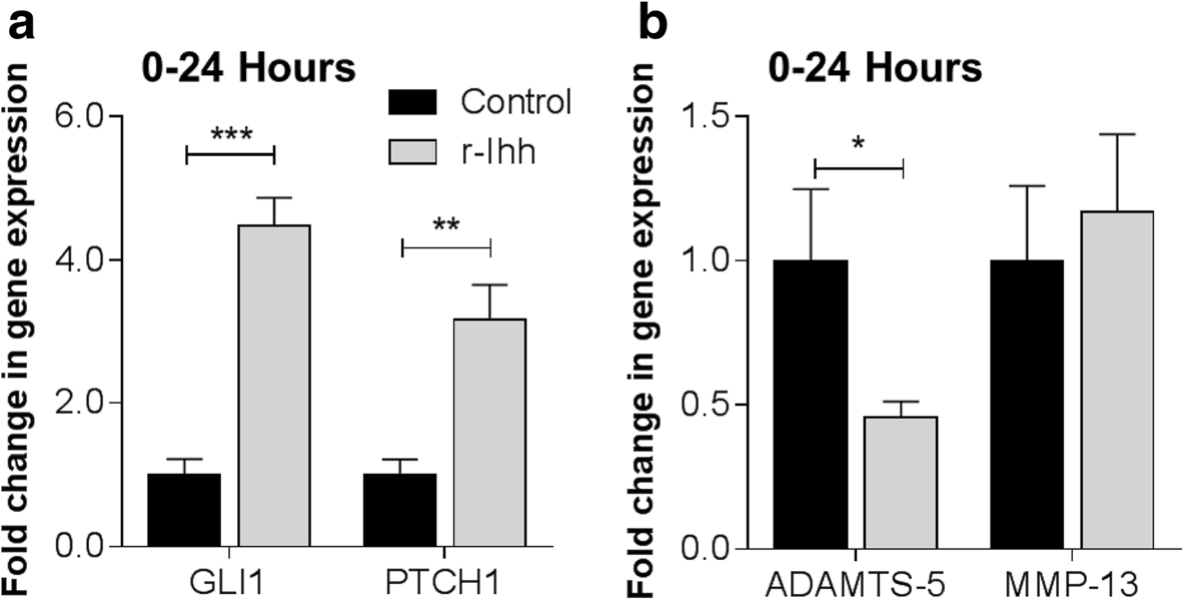
Hedgehog pathway activation does not increase the expression of a disintegrin and matrix metalloproteinase with thrombospondin motif (*ADAMTS-5*) and matrix metalloproteinase-13 (*MMP-13*) in bovine articular chondrocytes. After 24 hours of stimulation with r-Ihh, changes in the gene expression of (**a**) *Gli1* and *Ptch1* and (**b**) *ADAMTS-5* and *MMP-13* were measured. Data are mean fold-change normalised to untreated controls with standard deviation *error bars* (n = 9 from three separate donors). *Statistically significant differences relative to untreated controls, *P <0.05, **P ≤0.01, and ***P ≤0.001. *r-Ihh* recombinant Ihh

Isolated primary bovine chondrocytes were stimulated with r-Ihh for 24 hours to assess the effects of hedgehog pathway activation on catabolic gene expression. Following r-Ihh treatment, *Gli1* and *Ptch1* expression was increased relative to the unstimulated control by a magnitude of 4.5-fold (*P* ≤0.001) and 3.2-fold (*P* ≤0.01) respectively (Fig. 1a). The expression of *MMP-13* was not significantly altered following r-Ihh treatment, however, a small but significant reduction in *ADAMTS-5* expression was observed (0.5-fold, *P* ≤0.01) (Fig. 1b). These results demonstrate that r-Ihh successfully activated the hedgehog pathway but did not upregulate catabolic gene expression.

### Hedgehog pathway modulation does not alter cartilage degradation in bovine cartilage explants

**Fig. 2:**
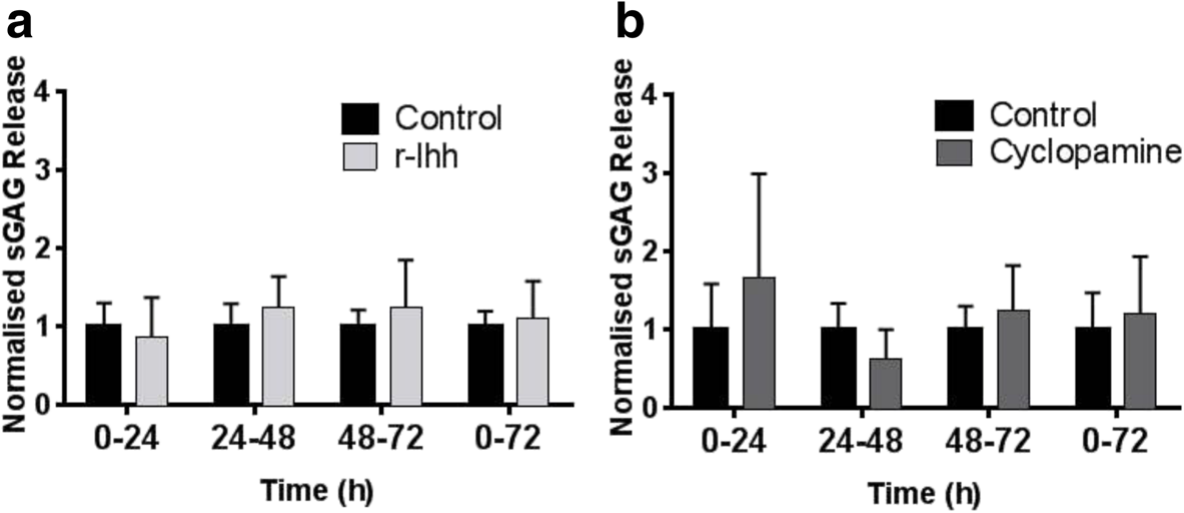
Hedgehog pathway modulation does not influence cartilage degradation in bovine explants. The sulphated glycosaminoglycans (*sGAG*) release for explants treated with r-Ihh (**a**) and cyclopamine (**b**) shown at 24-hour intervals and cumulatively over the 72-hour culture period as compared to untreated controls. All data are displayed as mean sGAG release, normalised to the untreated controls at the same time point, with standard deviation *error bars* (n = 9 from three separate donors). *r-Ihh* recombinant Ihh

The induction of mRNA does not always correlate with catabolic effects, therefore the effect of hedgehog pathway activation on cartilage degradation was directly investigated in bovine cartilage explants. Explants were treated with r-Ihh for 24, 48 and 72 hrs and cartilage degradation monitored by measuring the release of sGAG into the culture media (Fig. 2a). No significant change in sGAG release was observed at any time point relative to the untreated control. Consequently, there was no significant difference in the cumulative sGAG release at the end of the 72-hour culture period. Furthermore, no significant effects on sGAG release were observed when the treatment period was extended (Additional file 1: Figure S1).

The hedgehog pathway antagonist, cyclopamine, was similarly administered to bovine cartilage explants to examine the influence of endogenous basal hedgehog signalling on matrix catabolism. Cyclopamine significantly decreased sGAG release only at the 24–48 hour time point (*P <*0.05), at every other time point cyclopamine had no effect on sGAG release and there was no significant difference in cumulative sGAG release at the end of the 72-hour culture period (Fig. 2b). These results indicate that both hedgehog pathway activation by r-Ihh and inhibition by cyclopamine does not influence matrix catabolism in bovine cartilage. Cell viability analysis following treatment showed that the majority of cells are alive after the 72-day treatment period (Additional file 1: Figure S2A), with r-Ihh and cyclopamine treatment yielding similar cell viability as control (Additional file 1: Figure S2b).

### Hedgehog pathway activation has no effect on ADAMTS-5 or MMP-13 expression or matrix catabolism in human cartilage

**Fig. 3:**
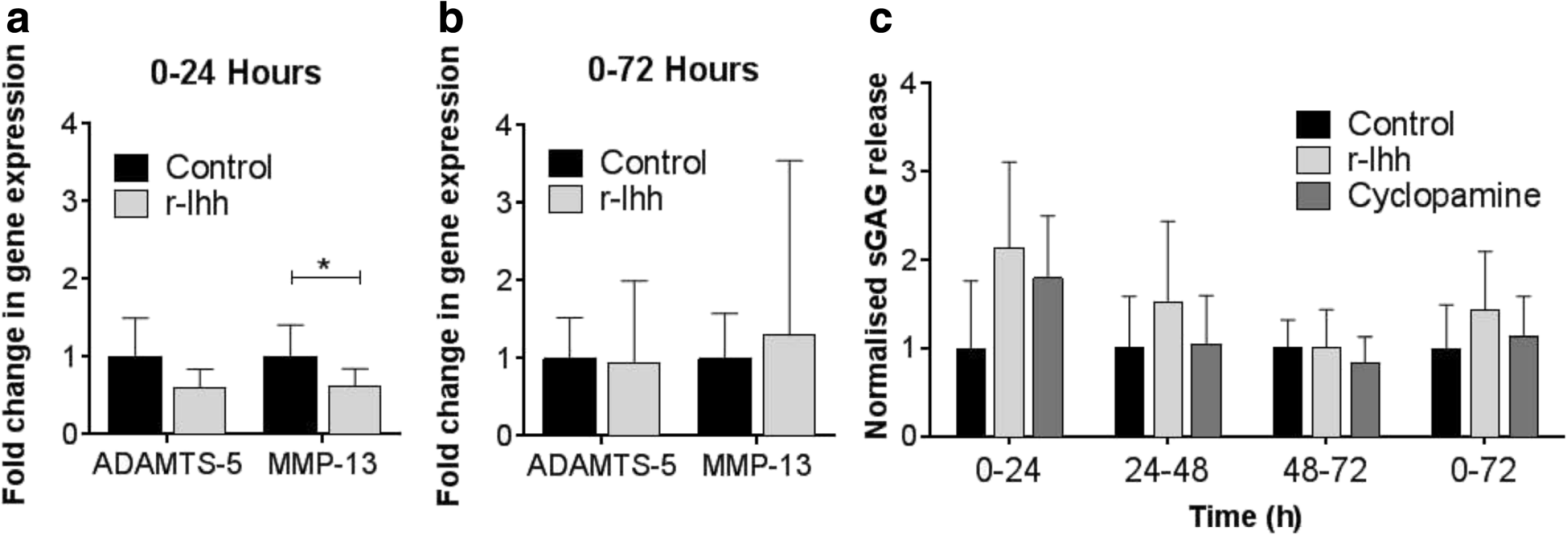
Hedgehog pathway activation has no effect on a disintegrin and matrix metalloproteinase with thrombospondin motif-5 (*ADAMTS-5*) and matrix metalloproteinase-13 (*MMP-13*) gene expression or on sulphated glycosaminoglycans (*sGAG*) release in human articular cartilage. Changes in the gene expression of *ADAMTS-5* and *MMP-13* in isolated human articular chondrocytes are shown after 24 hours (**a**) and 72 hours (**b**) of stimulation with recombinant Indian hedgehog (*r-Ihh*) compared to controls. Data are mean fold-change (n = 12 from three separate donors). **c** sGAG release for untreated controls, r-Ihh, and cyclopamine at 24-hour intervals over the 72-hour culture period, and cumulatively over the 72-hour culture period. Data are mean sGAG release, normalised to the untreated controls from the same donor tissue at the same time point, with standard deviation *error bars* (n = 6). *Statistically significant difference between groups and their respective untreated controls, *P <0.05, **P ≤0.01, and ***P ≤0.001

The above studies were repeated using human chondrocytes and cartilage explants to examine whether the absence of a catabolic response to r-Ihh was due to the use of bovine tissue. In isolated primary human chondrocytes, r-Ihh stimulation for either 24 or 72 hours did not significantly alter *ADAMTS-5* gene expression relative to the untreated control (Fig. 3a and b). A significant 0.6-fold reduction in *MMP-13* expression was observed at 24 hours (*P <*0.05, Fig. 3a), however, by 72 hours this was no longer significantly different to the control group (Fig. 3b). Neither r-Ihh nor cyclopamine significantly altered sGAG release in human cartilage explants (Fig. 3c). Similar to bovine tissue, these results suggest that hedgehog signalling plays a minimal role in modulating extracellular matrix catabolism in human articular cartilage.

### Hedgehog pathway modulation does not affect IL-1β-induced cartilage degradation in bovine cartilage explants

**Fig. 4:**
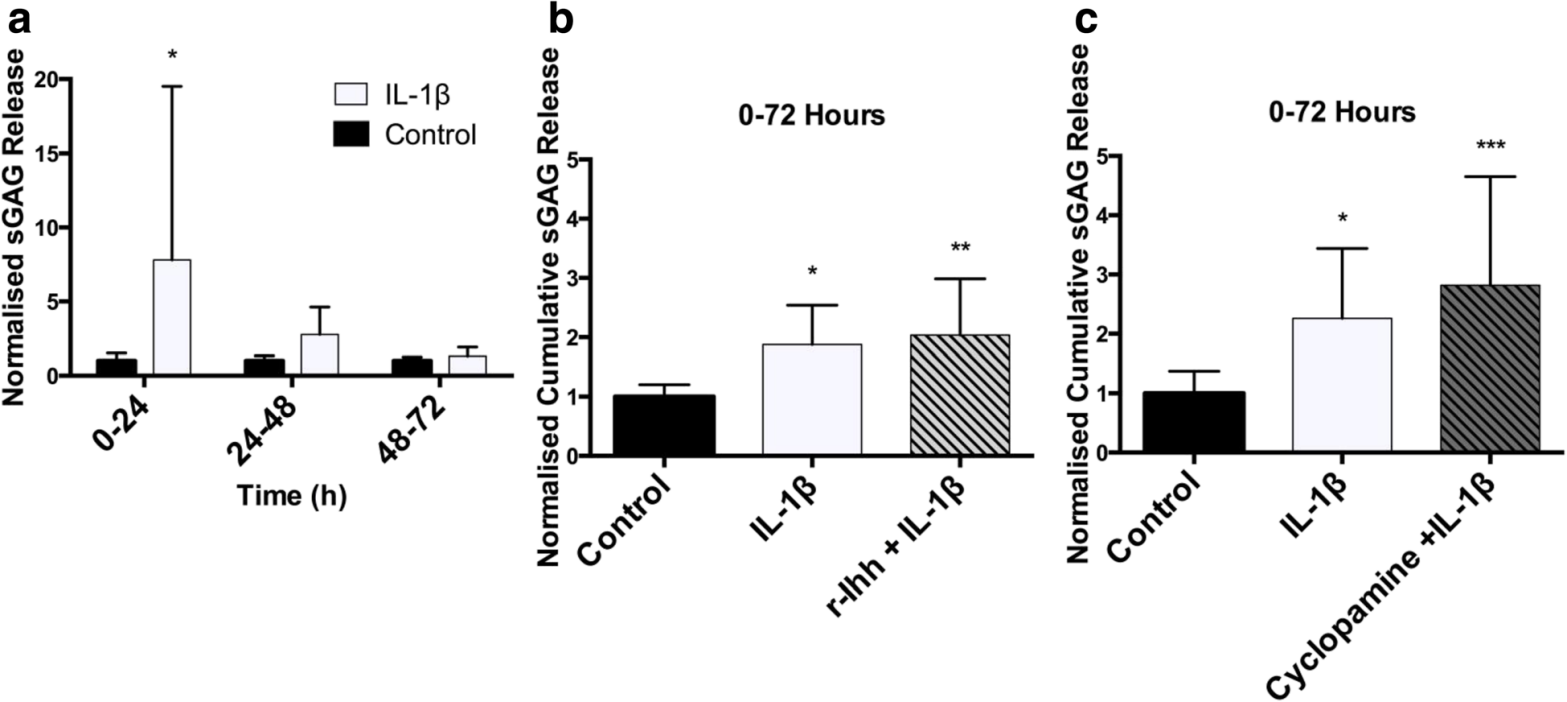
Hedgehog pathway modulation does not affect cartilage degradation in IL-1β-treated explants. **a** Changes in cumulative sulphated glycosaminoglycans (*sGAG*) release following treatment with IL-1β, from 0 to 24 hours, in bovine articular cartilage explants are shown over the 72-hour culture period. **b** The mean cumulative sGAG release at 72 hours for explants treated with IL-1β in the presence or absence of recombinant Indian hedgehog (*r-Ihh*) is displayed, compared to untreated controls. **c** Mean cumulative sGAG release at 72 hours for explants treated with IL-1β in the presence or absence of cyclopamine, compared to untreated controls. All data are mean sGAG release, normalised to untreated controls from the same animal at the same time point, with standard deviation *error bars* (n = 9 from three separate donors). *Statistically significant difference between the treatment groups and their respective untreated control, *P <0.05, **P ≤0.01, and ***P ≤0.001

Next, we sought to examine whether the previously reported degradative effects of hedgehog signalling were due to the presence of inflammatory cytokines in the OA microenvironment. Bovine cartilage explants were treated with 2.5 ng/mL IL-1β treatment for 24 hours. IL-1β treatment resulted in a significant increase in sGAG release over the 72-hour culture period compared to untreated controls (*P <*0.01) (Fig. 4c). Following the 24-hour IL-1β treatment, explants displayed a 7.8-fold increase in sGAG compared to untreated controls (*P <*0.05) (Fig. 4a). Thereafter, sGAG release decreased at each subsequent time point, with IL-1β-treated explants displaying 2.8-fold and 1.3-fold increases relative to controls at 48 and 72 hours, respectively, as the effects of the single dose treatment wore off. To investigate whether IL-1β influences the effect of hedgehog pathway activation on cartilage degradation, we evaluated the effects of r-Ihh and cyclopamine on sGAG release using this inflammatory model. The release of sGAG over 72 hours was not significantly modulated by the addition of r-Ihh (Fig. 4b) or cyclopamine (Fig. 4c) compared to IL-1β treatment alone. Additional antagonists, KAAD-cyclopamine, SANT-1, and GANT61, all showed a similar trend with no significant change in IL-1β-induced sGAG release at 72 hours (Additional file 1: Figure S3). Taken together, these results indicate that the presence of IL-1β in this in vitro model of inflammatory arthritis does not modify the catabolic response to hedgehog pathway activation.

### IL-1β suppresses hedgehog pathway activation in bovine articular chondrocytes

**Fig. 5:**
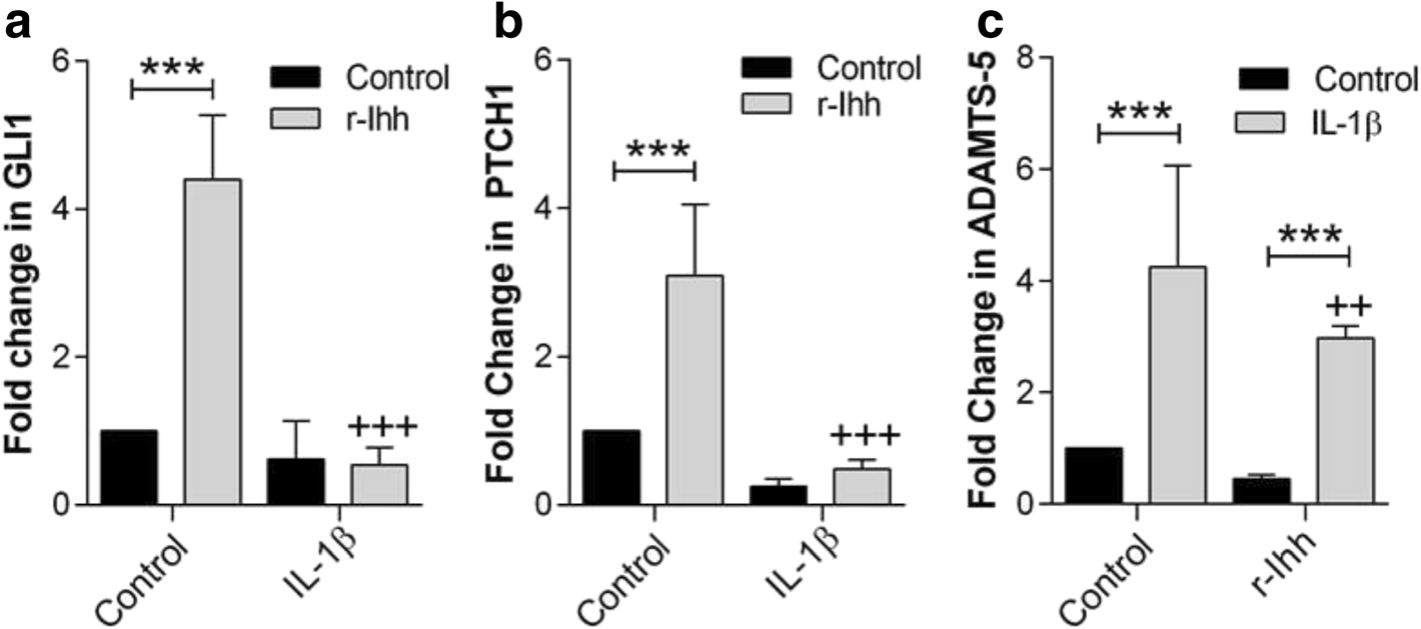
IL-1β reduces hedgehog pathway activation in response to recombinant Indian hedgehog (*r-Ihh*). After 24 hours of treatment, in bovine articular chondrocytes, IL-1β prevents upregulation in *GLI1* (**a**) and *PTCH1* (**b**) expression in response to r-Ihh treatment. **c** Treatment with IL-1β similarly repressed r-Ihh upregulation of a disintegrin and matrix metalloproteinase with thrombospondin motif (*ADAMTS-5*) expression but did not completely abolish this effect. *Statistically significant difference between the r-Ihh-treated samples and the untreated control; ^+^statistically significant difference between the r-Ihh/IL-1β-treated samples and the r-Ihh-treated samples (n = 9 from three separate donors), ***P ≤0.001, ^++^ *P* ≤0.01, and ^+++^ *P* ≤0.001

Finally, we examined whether IL-1β influenced the activation of hedgehog signalling in response to the addition of r-Ihh (Fig. 5). Treatment of isolated bovine chondrocytes with 10 ng/mL IL-1β inhibited r-Ihh-induced *Gli1* (Fig. 5a) and *Ptch1* (Fig. 5b) gene expression. Conversely, we also observed that activation of hedgehog signalling weakly downregulates IL-1β-induced *ADAMTS-5* expression (Fig. 5c). These results suggest there is molecular crosstalk between the hedgehog and IL-1β signalling pathways in chondrocytes.

## Discussion

In this study, we examined the effects of hedgehog signalling on *ADAMTS-5* and *MMP-13* gene expression and cartilage degradation in isolated chondrocytes and cartilage tissue, and in a well-established in vitro explant model of inflammatory arthritis [19]. The efficacy of this IL-1β-treatment protocol has been proven in our previous work, which showed significant effects of IL-1β-treatment on a number of parameters such as PGE_2_, NO, matrix degradation and biomechanical changes [19, 27–29]. Our work is the first to illustrate that ligand-activation of chondrocyte hedgehog signalling is unable to stimulate the expression of the pro-catabolic genes *ADAMTS-5* and *MMP-13* or directly induce cartilage matrix breakdown. We also demonstrated that even in IL-1β treated explants, the activation or inhibition of hedgehog signalling cascade does not yield any significant effects on cartilage matrix degradation.

As these studies are focused on examining ligand-induced hedgehog signalling, the basal levels of hedgehog signalling was not measured in these studies. We have, however, shown the induction of hedgehog signalling via Ihh-induced upregulation of Gli1 and Ptch1 (Fig. 1a and Fig. 5a and b). Despite successful activation of the hedgehog pathway, the expression of *ADAMTS-5* and *MMP-13* was not significantly altered by r-Ihh treatment in bovine or human articular chondrocytes. This lack of catabolic response to hedgehog signalling is an apparent contrast to previous studies [26, 28]. These differences, however, were not due to the loss of cellularity, as shown by cell viability analysis, which was approximately 70 % in all groups including controls at the end of the 72-hour treatment period. In previous studies, hedgehog pathway activation was shown to upregulate the expression of these catabolic genes downstream of the transcription factor Runx2 [5]. It is therefore possible that the 24-hour treatment used in the current study was not sufficient to regulate gene expression. However, *ADAMTS-5* and *MMP-13* expression were not significantly influenced by r-Ihh in human articular chondrocytes following longer treatment for 72 hours either. Hedgehog signalling also failed to stimulate matrix catabolism in bovine cartilage explants. Similar results were obtained for human cartilage explants, which, however, were taken from a single donor and therefore can only be used as validation for bovine and isolated chondrocyte studies. Overall, these data indicate that hedgehog pathway activation by itself does not stimulate cartilage degradation.

Our results may appear to be in contrast to previous studies linking the hedgehog pathway to *ADAMTS-5* and *MMP-13* expression. For example, Lin et al. demonstrated that r-Ihh stimulation in human cartilage in OA increases the expression of both *ADAMTS-5* and *MMP-13* [26]. Similarly, Zhou et al. demonstrated that surgically induced OA in Ihh-deleted mice results in the decreased expression of *MMP-13* [27]. However, to the best of our knowledge, this is the first study to examine the effect of Ihh on *ADAMTS-5* and *MMP-13* expression in healthy chondrocytes, as opposed to the OA models used in previous studies where Ihh signalling deregulated the expression of catabolic enyzmes [4–6, 9, 15, 17, 18, 30, 31]. Ihh signalling has been described as “context-dependent” such that it inhibits or promotes chondrocyte hypertrophy depending on the levels of parathyroid hormone-related protein present [32]. It is thus possible that Ihh signalling may be influenced by its interactions with the complex biochemical milieu within cartilage in OA. It is possible however that these alterations in hedgehog signalling may have long-term effects that are not measurable in the time course used in these studies. These include potential effects on chondrocyte fate, including an eventual development of chondrocyte hypertrophy and subsequent upregulation in catabolic enzymes and the development of osteoarthritic phenotype.

In healthy cartilage, Runx2 expression is inhibited directly by HDAC4 and indirectly by transforming growth factor-β (TGF-β), via Smads 2 and 3. Therefore two plausible candidates for interaction are histone deacetylase 4 (HDAC4) and TGF-β [17, 33]. The inhibitory effects of these proteins on Runx2 may prevent Ihh-induced expression of Runx2 in healthy cartilage, which would explain the lack of Ihh-induced *ADAMTS-5* and *MMP-13* upregulation observed in the current study. In OA, the levels of HDAC4 are decreased, relieving its inhibition of Runx2. Similarly in OA, TGF-β activates Smads 1, 5, and 8, which promote Runx2 expression [17, 33]. It is in this context that Ihh may be able to contribute to the upregulation of Runx2 and subsequent *ADAMTS-5*- and *MMP-13*-mediated cartilage degradation in the joints in OA.

In this study, we also examined the influence of hedgehog pathway activation in a well-established in vitro bovine explant model of inflammatory arthritis [34]. We have previously shown that IL-1β induces the trafficking of key signalling proteins onto primary cilia with an associated increase in cilia length [27–29]. As hedgehog signalling in chondrocytes also requires ciliary trafficking, we hypothesised that IL-1β synergises the catabolic response to hedgehog ligand through modulation of the primary cilium. This model is based on the pro-inflammatory cytokine, IL-1β, which is upregulated in inflammatory arthritis and causes cartilage degradation [19, 20, 35, 36]. Although there are numerous factors that contribute to the development of OA, including pathological increases in other cytokines, injurious mechanical loading, increased wnt signalling etc., it is challenging to incorporate all these factors into a single in vitro model. Bovine cartilage explants were treated with 2.5 ng/mL of IL-1β from 0–24 hours and then treated with normal chondrocyte media for the remainder of the 72-hour culture period (Fig. 4). While higher than the long-term pathological concentrations of IL-1β measured within the joints in OA [34, 37], the dosing strategy used in the current study aimed to strike a balance between having a sufficiently high IL-1β dose to induce detectable levels of matrix degradation over a short period of time without overshadowing the possible catabolic effects of hedgehog signalling. IL-1β treatment increased cartilage degradation throughout the 72-hour culture period compared to untreated controls; this was not influenced by treatment with r-Ihh. In addition, the hedgehog antagonists, cyclopamine, KAAD-cyclopamine, SANT-1, and GANT61, did not display any significant inhibition of IL-1β-induced cartilage damage, despite previous reports suggesting a potential therapeutic role for hedgehog blockade in arthritis [4–6]. Thus, these data show no synergistic interaction between the catabolic effects of hedgehog signalling and IL-1β.

Finally, we showed that in IL-1β-treated chondrocytes, the upregulation of *Gli1* and *Ptch1* in response to r-Ihh is greatly reduced. We also showed that IL-1β induces an upregulation in ADAMTS5 expression, which was significantly reduced but not abolished by Ihh-treatment. We suggest that this is due to the increase in primary cilia length with IL-1β treatment [28] as our recent studies show an inverse relationship between cilia length and hedgehog signalling [9, 38]. The mechanism for reduced hedgehog signalling may be due to reduced intraflagellar transport [28, 39] and changes in ciliary trafficking [29, 40].

## Conclusion

Previous studies report that an aberrant upregulation of hedgehog signalling occurs in OA, leading to cartilage degradation [4–6]. However, this study showed for the first time that in healthy cartilage in vitro, hedgehog pathway activation by itself does not cause cartilage degradation. We therefore examined whether the presence of inflammatory cytokines stimulated hedgehog-induced catabolism. However, even in the presence of IL-1β, neither hedgehog agonists nor antagonists had any effect on matrix degradation. Thus, we suggest that the reported role of hedgehog signalling in matrix catabolism in OA must be due to a complex interaction with other pathological factors or more long-term effects on chondrocyte fate. Importantly, we demonstrated that IL-1β suppresses hedgehog pathway activation by r-Ihh therefore any possible effects of hedgehog signalling in OA are likely to be downregulated by the presence of inflammatory cytokines. We conclude that chondrocyte hedgehog signalling is not catabolic in otherwise healthy cartilage and is in fact downregulated by inflammatory cytokines, such that any role for hedgehog is extremely context-dependent.

## Additional file

Additional file 1:
**Figure S1.** Hedgehog pathway activation over 6 days does not influence cartilage degradation. Changes in sulphated glycosaminoglycans (*sGA*G) release following treatment with IL-1β or recombinant Indian hedgehog (*r-Ihh*), in bovine articular cartilage explants, over a 6-day culture period. Although IL-1β increased sGAG release into the media over the 6-day culture period, there was no upregulation in sGAG in response to r-Ihh treatment (n = 6 from three separate donors). **Figure S2.** Treatment with r-Ihh or cyclopamine had no effect on chondrocyte viability. **a** Representative fluorescence microscopy image showing live cells (calcein AM/*green*) and dead cells (ethidium homodimer/*re*d) within a cartilage explant. *Scale bar* represents 50 μm. *Arrows* indicate the articular surface. **b** Percentage cell viability. Values represent mean with *error bars* showing standard deviations (n = 9 from three separate donors). **Figure S3.** Hedgehog pathway antagonists KAAD-cyclopamine, SANT-1, and GANT61 do not inhibit cartilage degradation in IL-1β-treated explants. Following treatment with IL-1β for 24 hours, bovine cartilage explants exhibit an increase in cumulative sGAG release after 72 hours compared to untreated controls, however, the hedgehog antagonists KAAD-cyclopamine, SANT-1, and GANT61 did not significantly modulate the effects of IL-1β. All data are mean sGAG release, normalised to untreated controls from the same animal at the same time point (n = 9 from three separate donors). *Statistically significant difference, **P* < 0.05, ***P* ≤0.01, and ****P* ≤0.001. (DOCX 1215 kb)

## Abbreviations

ACAN: aggrecan gene
ADAMTS: a disintegrin and matrix metalloproteinase with thrombospondin motif
BSA: bovine serum albumin
COL2A1: type II collagen gene
COX-2: prostaglandin E_2_ producing enzyme
DMEM: Dulbecco’s modified Eagle’s medium
DMMB: dimethymethylene blue
FBS: foetal bovine serum
HDAC4: histone deacetylase 4
Ihh: Indian hedgehog
IL-1β: interleukin-1β
iNOS: nitric oxide producing enzyme
MMP: matrix metalloproteinase
NO: nitric oxide
OA: osteoarthritis
PBS: phosphate-buffered saline
PGE_2_: prostaglandin E_2_
Ptch1: patched 1
r-Ihh: recombinant Ihh
Runx2: runt-related transcription factor 2
sGAG: sulphated glycosaminoglycans
Smo: Smoothened
TGFβ: transforming growth factor β
