# Supplementary figures and images for: Hedgehog signalling does not stimulate cartilage catabolism and is inhibited by Interleukin-1β
Source: Arthritis Res Ther. 2015 Dec 24;17:373. doi: 10.1186/s13075-015-0891-z (PMC4718026; doi:10.1186/s13075-015-0891-z)

**
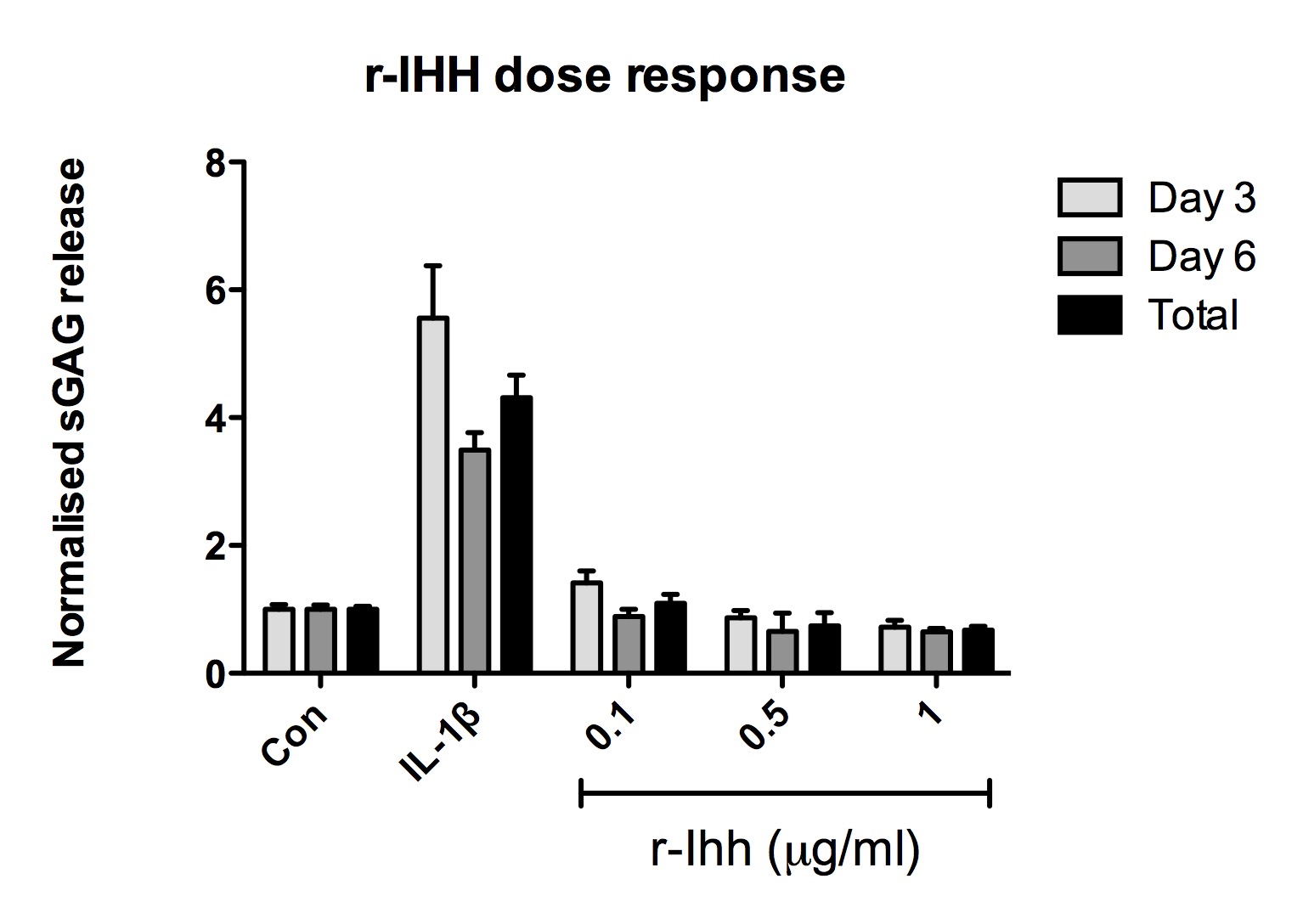
**

**Figure S1**

**
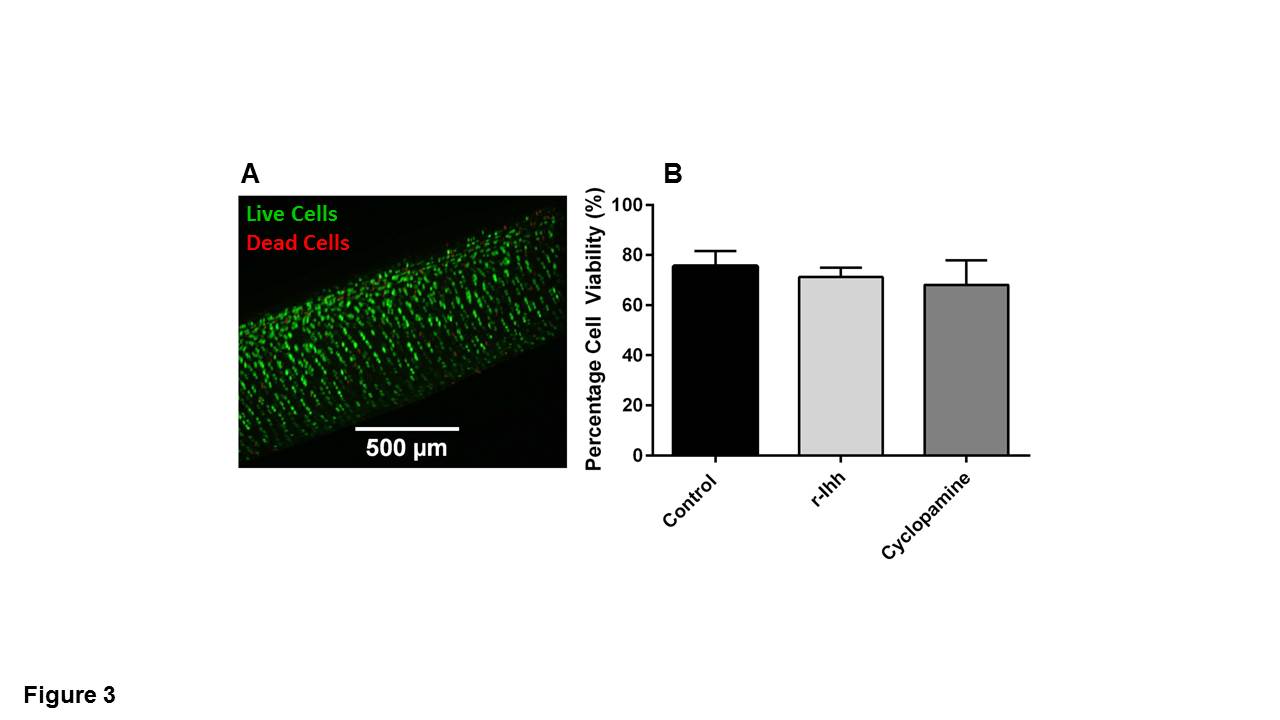
**

**Figure S2**

**
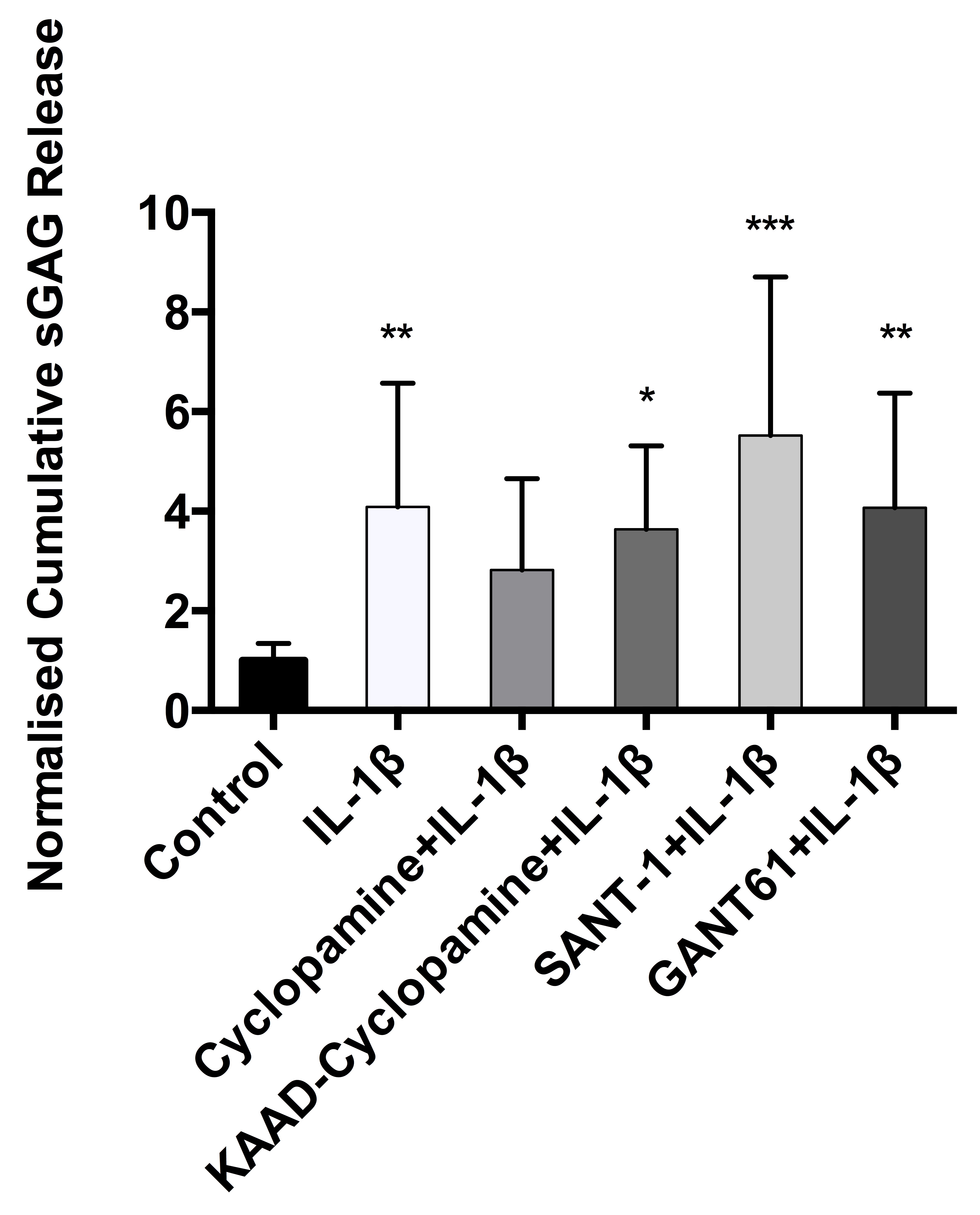
**

**Figure S3**

Supplement: Additional file 1: — Figure S1. Hedgehog pathway activation over 6 days does not influence cartilage degradation. Changes in sulphated glycosaminoglycans (sGAG) release following treatment with IL-1β or recombinant Indian hedgehog (r-Ihh), in bovine articular cartilage explants, over a 6-day culture period. Although IL-1β increased sGAG release into the media over the 6-day culture period, there was no upregulation in sGAG in response to r-Ihh treatment (n = 6 from three separate donors). Figure S2. Treatment with r-Ihh or cyclopamine had no effect on chondrocyte viability. a Representative fluorescence microscopy image showing live cells (calcein AM/green) and dead cells (ethidium homodimer/red) within a cartilage explant. Scale bar represents 50 μm. Arrows indicate the articular surface. b Percentage cell viability. Values represent mean with error bars showing standard deviations (n = 9 from three separate donors). Figure S3. Hedgehog pathway antagonists KAAD-cyclopamine, SANT-1, and GANT61 do not inhibit cartilage degradation in IL-1β-treated explants. Following treatment with IL-1β for 24 hours, bovine cartilage explants exhibit an increase in cumulative sGAG release after 72 hours compared to untreated controls, however, the hedgehog antagonists KAAD-cyclopamine, SANT-1, and GANT61 did not significantly modulate the effects of IL-1β. All data are mean sGAG release, normalised to untreated controls from the same animal at the same time point (n = 9 from three separate donors). *Statistically significant difference, *P < 0.05, **P ≤0.01, and ***P ≤0.001. (DOCX 1215 kb) [file 13075_2015_891_MOESM1_ESM.docx]
